# Exoskeleton Walk Training in Paralyzed Individuals Benefits From Transcutaneous Lumbar Cord Tonic Electrical Stimulation

**DOI:** 10.3389/fnins.2020.00416

**Published:** 2020-05-25

**Authors:** Elena Y. Shapkova, Elena V. Pismennaya, Dmitriy V. Emelyannikov, Yury Ivanenko

**Affiliations:** ^1^The Spinal Center of Saint-Petersburg State Research Institute of Phthisiopulmonology, Saint Petersburg, Russia; ^2^Institute of Translational Biomedicine, Saint Petersburg State University, Saint Petersburg, Russia; ^3^Institute of Mechanics, Lomonosov Moscow State University, Moscow, Russia; ^4^IRCCS Santa Lucia Foundation, Rome, Italy

**Keywords:** spinal cord injury, exoskeleton, spinal cord electrical stimulation, spasticity, locomotion, neurorehabilitation

## Abstract

In recent years, advanced technologies featuring wearable powered exoskeletons and neuromodulation of lumbosacral spinal networks have been developed to facilitate stepping and promote motor recovery in humans with paralysis. Here we studied a combined effect of spinal cord electrical stimulation (SCES) and exoskeleton walk training (EWT) during an intensive 2-week rehabilitative protocol in spinal cord injury individuals (*n* = 19, American Spinal Injury Association Impairment Scale (AIS) A-11, B-5, C-3). The purpose of this study was to evaluate the compatibility of methods and to explore the main effects of combined SCES and EWT. All participants had a chronic state of paralysis (1–11 years after trauma). In addition, in the control group (*n* = 16, AIS A-7, B-5, C-4), we performed EWT without SCES. For EWT, we used a powered exoskeleton (ExoAtlet), while stability was assisted by crutches, with automatic arrest of stepping if excessive torques were detected. SCES was applied to the level of the mid-lumbar cord over the Th12 vertebra at 1 or 3 pulses/s (4 individuals with severe spasticity were also stimulated in an anti-spastic mode 67 pulses/s). The vertical component of the ground reaction force was recorded using the F-Scan system at the onset and after training with SCES. EWT with SCES significantly increased the foot loading forces, could decrease their asymmetry and 8 out of 19 subjects improved their Hauser Ambulation Index. The anti-spastic mode of stimulation also allowed individuals with severe spasticity to walk with the aid of the exoskeleton. Participants reported facilitation when walking with SCES, paresthesia in leg muscles and new non-differential sensation of passive motion in leg joints. Neurological examination showed an increase of tactile (7) and/or pain (7) sensation and an increase of the AIS motor scale in 9 individuals, including both incomplete and complete paralysis. Improvements in the neurological scores were, however, limited in the control group (EWT without SCES). The results suggest that SCES may facilitate training and walking in the exoskeleton by activating the locomotor networks and augmenting compensative sensitivity.

## Introduction

In the last decade, interest has grown around the use of exoskeleton-induced walk for the rehabilitation of paralyzed patients. Exoskeletons (Exo) have been shown to enable over-ground weighted walking and gait training for the mobility impaired persons, particularly for individuals with spinal cord injury (SCI) (del-Ama et al., 2012; Sylos-Labini et al., 2014; Onose et al., 2016). Single case reports of supplemental functional stimulation of leg muscles (Ekelem and Goldfarb, 2018) and spinal cord neuromodulation (Gad et al., 2017) during Exo-assisted gait have shown improved kinematics and muscle activity patterns in motor complete SCI individuals. Nevertheless, the efficacy of Exo training for individuals with SCI is still under discussion (Contreras-Vidal et al., 2016; Fisahn et al., 2016; Scivoletto et al., 2019).

Earlier works suggested a beneficial effect of spinal cord electrical stimulation on activating spinal locomotor circuits (Dimitrijevic and Larsson, 1981; Shapkov et al., 1996) and controlling spinal spasticity (Richardson and McLone, 1978; Siegfried et al., 1978; Vodovnik et al., 1984). The effectiveness of different stimulation parameters has also been explored (Iwahara et al., 1992; Dimitrijevic et al., 1998; Shapkova, 2004). In our clinic, for more than 25 years we have been developing a method of transcutaneous spinal cord electrical stimulation (SCES) to activate the neuronal locomotor networks in paralyzed individuals (Shapkov et al., 1996; Shapkova and Schomburg, 2001; Shapkova, 2004). In particular, we demonstrated that application of tonic (either epidural or transcutaneous) electrical stimulation at the mid-lumbar enlargement (about L3-L5 spinal segments, Th12 vertebrae) could evoke well-coordinated, alternating, step-like movements in individuals with complete and incomplete paralysis (Shapkova, 2004). Because the frequency of “stepping” was generally independent of the SCES frequency and rhythmic movements could continue for many cycles after the end of stimulation, the induced activity was considered to be centrally generated by activating the spinal pattern generation circuitry. Using such approaches, many researchers have put significant effort into assessing and modulating the functional state of the spinal locomotor circuits in humans (Mayr et al., 2016; Ivanenko et al., 2017; Gill et al., 2018; Hofstoetter et al., 2018; Taccola et al., 2018; Wagner et al., 2018; Megía García et al., 2020). Similar concepts have been developed using animal models (Musienko et al., 2012; Lavrov et al., 2015; Wenger et al., 2016). Epidural stimulation is typically more localized and requires less current of stimulation, while transcutaneous stimulation is non-invasive, although both techniques seem to largely activate the posterior root fibers (even if it is difficult to fully rule out that the stimulation is also acting on the neural circuitry in the cord) (see Hofstoetter et al., 2018 for a review). Since common neural structures are activated by epidural and transcutaneous lumbar spinal cord stimulation with a similar effect (Hofstoetter et al., 2018), the use of non-invasive SCES has become widely spread over for both basic and clinical research (Shapkova, 2004; Gerasimenko et al., 2015; Hofstoetter et al., 2015; Mayr et al., 2016; Solopova et al., 2017; Barroso et al., 2019).

In the context of looking for adaptive therapies for entraining the spinal locomotor circuitry, it may be of interest to explore the effect of combined interventions since the efficiency of SCES at rest might be limited without a concurrent locomotor function training, which may entrain activity-dependent plasticity of spinal neuronal networks. A combination of two highly intensive methods, such as powered exoskeleton walk training (EWT) and spinal cord electrical stimulation, stimulating afferent and efferent inputs within a functional task, may lead to effective neural plasticity that provides a basis for recovery. Therefore, the purpose of this study was to evaluate the compatibility of methods and to explore the main effects of combined SCES and EWT. To this end, thirty five participants with complete and incomplete SCI were enrolled in this exploratory study and were grouped according to the stimulation (frequency of SCES) method.

## Materials and Methods

### Participants

**FIGURE 1:**
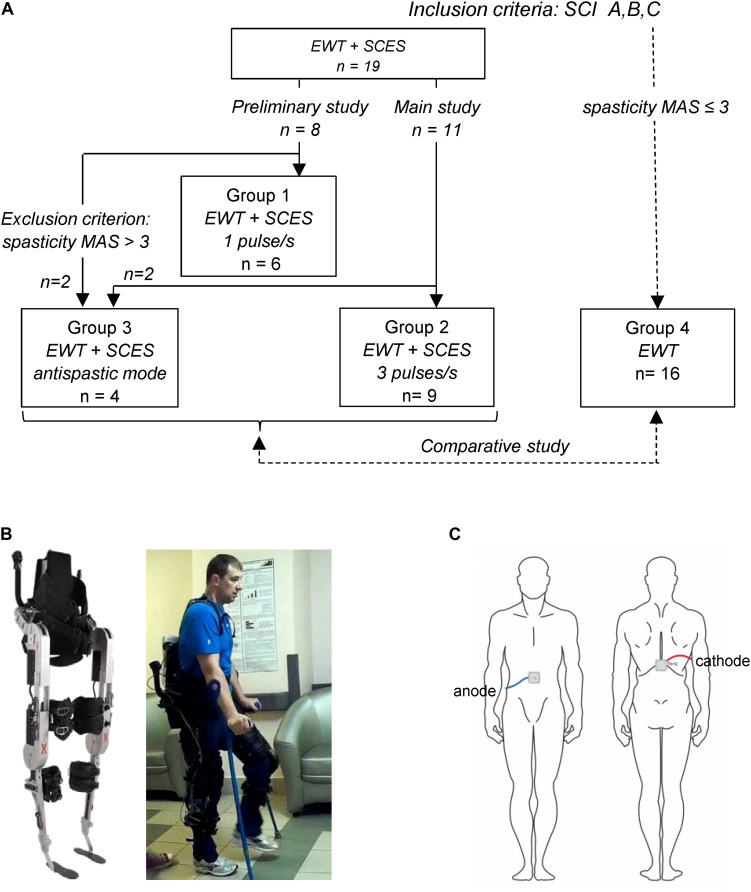
Experimental setup. **(A)** General scheme of participant groups and corresponding inclusion criteria. **(B)** Wearable powered exoskeleton (left) and an example of walking in the exoskeleton in the SCI participant (P12, AIS B, 11 years post-injury, see Table 1) without the assistance of the physiotherapist at the end of 2-week training (written informed consent was obtained from the individual for the publication of this image). The exoskeleton is attached to the wearer at five main locations: footplate, shank, thigh, pelvis, and torso. In each leg, hip flexion/extension and knee flexion/extension) are powered by actuators, providing appropriate kinematics of joint angle motion, 8 different walking modes and 3 speeds (corresponding to the cycle duration of *T* = 3, 4, or 5 s), and an automatic arrest of stepping if excessive torques are detected. **(C)** Schematic illustration of electrode placements.

Thirty five adults with traumatic chronic SCI participated in this study (10♀, 25♂, post-trauma period from 1 to 11 years). Figure 1A illustrates a general scheme of participants’ groups and Table 1 shows the characteristic of participants. The criteria for inclusion were: SCI with paralysis estimated as class A, B, or C of American Spinal Injury Association Impairment Scale (AIS); decompression and stabilization of spine in anamnesis, confirmed by MRI and/or CT data; post-trauma period more than 12 mo; age within 18–55 years; ability to stand for 30 min without pathological orthostatic reactions; high motivation to motor recovery; written informed consent to participate in the study. The exclusion criteria were: low extremities’ bone fractures within post-trauma period; the thrombosis in the vessels of lower extremities (ultrasound or Doppler data); decubitus or maceration of the skin; other neurological diseases. All subjects gave written informed consent. This study was reviewed and approved by the Ethics Committee of the Saint-Petersburg State Research Institute of Phthisiopulmonology and carried out in accordance with the Declaration of Helsinki.

**TABLE 1:** General information about the participants of the study.

Patient	Gender	Age, years	Level of spine damage	Level of spinal cord damage	Duration of paralysis, yrs	Rivermead Mobility Index	Barthel Index	AIS	AIS motor before/after training	AIS sensory LT before/after training	AIS sensory PP before/after training	Muscle tone, for spasticity: MAS before and after training	# of training sessions	# of training sessions with SCES	Total training duration, min	Averaged session duration, min	Walk duration, min	Max non-stop walk, min	Previous experience	Hauser Ambulation Index (0–9) before/after training
**Group 1** (1 pulse/s SCES)																				

P1	Ì	37	Th9	D9	1.5	4	65	A	0/0	60/60	60/60	Spastic R2/2, L2/2	15	9	872	58	380	8.7	SCES	**8/7**
P2	F	33	Th4-5	D4	4.8	3	30	A	0/0	**44/48**	**46/48**	Normal R0/0, L0/0	13	7	625	48	262	5.3	–	8/8
P3	Ì	21	Th5-7	D4	2	0	25	A	1/1	**40/43**	43/43	Spastic R2/3, L2/3	13	7	785	60	265	9.5	–	8/8
P4	Ì	37	Th12-L1	D10-12	2.5	4	75	C	19/19	**87/90**	**87/90**	Low	12	8	743	62	394	15.2	SCES	**7/6**
P5	Ì	38	Th10-12	D10	6	4	60	A	0/0	64/64	64/64	Low	12	8	616	51	315	21.5	–	8/8
P6	F	55	Ñ6-7	C8	10	3	60	B	0/0	74/74	42/42	Low	8	7	294	37	166	3.5	–	**9/8**
mean ± SD													13	8	656 ± 202	53 ± 10	297 ± 85	10.6 ± 6.7		

**Group 2** (3 pulses/s SCES)																				

P7	Ì	38	Th10	D9	2.5	3	65	A	**0/1**	62/62	64/64	Spastic R1/0, L1/0	10	9	432	43	302	37.6	EXO, SCES	**7/6**
P8	F	34	Th5	D4	6	3	60	A	**0/1**	44/44	44/44	Normal R0/0, L0/0	10	8	428	43	278	37.2	EXO, SCES	8/8
P9	Ì	20	Th5-6	D4	11	3	65	À	6/6	78/78	66/66	Low with distal clonus	10	9	395	40	271	21.5	SCES	8/8
P10	Ì	21	Th5	D5	4	4	55	A	**0/1**	44/44	**44/45**	Spastic R3/3, L3/2	9	9	348	39	205	25.4	EXO, SCES	8/8
P11	Ì	32	Th5-6	D4	4.5	2	60	À	0/0	44/44	44/44	Low with distal clonus	10	8	415	42	274	23.8	SCES	8/8
P12	Ì	37	Th12, L1-2	L2	11	3	75	B	**6/9**	107/107	103/103	Low	7	7	460	66	380	185.0	EXO	8/8
P13	F	27	Th10	D11	3	7	75	B	**0/3**	**89/91**	**91/93**	Low with distal clonus	10	9	404	40	286	40.8	EXO	**7/6**
P14	Ì	20	Th5-6	D7	2	7	90	B	0/0	58/58	58/58	Spastic R3/3, L2/2	7	6	223	33	137	18.4	EXO	**7/6**
P15	Ì	24	Th12	L2	1.5	7	65	C	**12/13**	**98/100**	**100/102**	Spastic R1/1, L1 + /1 +	9	7	226	25	157	21.9	EXO	**7/6**
mean ± SD (*excluding P12)													9	8	370 ± 88	41 ± 11	255 ± 76	28.3 ± 8.2*		

**Group 3** (67 + 3 pulses/s SCES)																				

P16	Ì	27	Th5-8	D6	9	3	50	A	0/0	**60/63**	**60/63**	Spastic R4/3, L4/3	11	8	766	70	278	8.15	–	8/8
P17	Ì	28	Th5-7	D5	1.5	3	40	Â	**0/3**	68/68	**56/58**	Spastic R4/3, L4/4	10	9	408	41	279	31.7	EXO, SCES	8/8
P18	Ì	31	Th7	D7	1	3	8	À	**0/2**	52/52	52/52	Spastic R4/3, L4/3	11	10	507	46	388	40.4	EXO, SCES	8/8
P19	Ì	32	C5-7, Th1	D3	4	4	75	C	**12/14**	**110/112**	75/75	Spastic R4/3, L4/3	9	8	308	34	204	14.2	SCES	**7/6**
mean ± SD													10	9	497 ± 197	48 ± 15	287 ± 76	23.6 ± 15.0		

**Group 4** (control, without SCES)																				

P20	M	36	Th7	D11	3	3	55	A	0/0	73/73	73/73	LOW with distal clonus	12	0	526	44	422	39	–	8/8
P21	M	28	Th12	L1	2	3	55	B	2/2	78/78	78/78	Spastic R1/1,L1/1	12	0	479	40	384	40	–	7/7
P22	M	44	Th12	L3	6	3	55	C	**17/18**	**88/90**	**90/92**	Spastic R2/2,L3/2	12	0	469	39	390	39	–	7/7
P23	M	33	Th12-L1	L2	11	4	85	C	16/16	94/94	92/92	Low	12	0	502	42	419	41	–	7/7
P24	F	20	Th5-6, Th9-10	D4	2	4	65	A	52/52	44/44	69/69	Normal R0/0,L0/0	12	0	581	48	381	24	–	8/8
P25	M	38	C7-Th1	D3	10	4	90	B	2/2	68/68	35/35	Spastic R3/3,L3/3	12	0	377	31	290	32	–	**7/6**
P26	M	38	Th12	D7	10	4	75	A	12/12	88/88	88/88	Low	12	0	462	39	400	42	–	**7/6**
P27	F	39	Th12	D12	10	3	45	A	0/0	74/74	72/72	Low	12	0	304	25	221	27	–	8/8
P28	M	44	Th12	L1	5	3	65	C	78/78	78/78	**63/67**	Spastic R1+/1+, L1+/1+	12	0	347	29	166	17	–	8/8
P29	F	27	Th3	D1	7	2	40	A	0/0	42/42	40/40	Spastic R3/3,L3/3	12	0	450	37	306	26	–	8/8
P30	F	18	Th12-L1	L1	5	3	65	B	6/6	78/78	78/78	Low	12	0	359	30	280	41	–	7/7
P31	F	21	Th11-L1	D10	4	3	75	B	0/0	72/72	64/64	Low	12	0	387	32	272	17	–	7/7
P32	F	27	Th7	D7	7	3	60	B	0/0	82/82	52/52	Spastic R3/3,L3/3	12	0	237	20	114	12	–	8/8
P33	M	49	Th7-9	D10	2	2	55	C	15/15	**72/82**	64/64	Spastic R3/3,L3/3	12	0	222	19	138	16	–	8/8
P34	M	41	Th6-7	D7	2	3	75	A	1/1	64/64	57/57	Spastic R1/1,L1/1	12	0	411	34	340	33	–	8/8
P35	M	30	Th8-9	D8	3	3	55	A	0/0	**58/60**	58/58	spastic R2/2,L0/2	12	0	437	36	285	14	–	8/8
mean ± SD													12	0	409 ± 100	34 ± 8	300 ± 100	28.8 ± 11.0		

AIS, American Spinal Injury Association Impairment Scale (LT, light touch; PP, pin-prick), MAS, Modified Ashworth Scale before/after training for both right (R) and left (L) sides. Previous experience of SCES refers to ∼2–3 weeks of SCES (∼1 h per day) in the supine position, ∼1 year prior to the study, to activate the spinal locomotor networks. Previous experience of EXO refers to advanced EXO-walkers (practice for ∼6–12 months) with no experience in SCES. Previous experience of “EXO, SCES” refers to experience in both SCES and EWT ∼1 year prior to this study. We marked in bold changes in neurological indicators (AIS) and in the Hauser Ambulation Index after training. *Patient (P12) was excluded from calculating the max non-stop walk (marked by the asterisk) because of his outstanding personal results (outlier).

Nineteen volunteers [4♀, 15♂, 31.2 ± 8.6 years (mean ± SD)] performed EWT with SCES (Groups 1–3). Fifteen participants had a lesion at thoracic level, two participants at thoracic-lumbar level, two participants at low-cervical level with partly/mostly saved functions of the arms. According to ASIA standards, neurological state of participants was estimated as class A (11), B (5), and C (3) (Table 1). The functional mobility of participants varied from 0 to 7 (3.7 ± 1.7, Rivermead Mobility Index), activities of daily living – from 8 to 90 (on average 57.8 ± 20, Barthel Index). For ambulation, all subjects used a wheelchair: ambulation was estimated as 8 and 9 by the Hauser Index (restricted to wheelchair) in 13 individuals, and as 7 (walking limited to several steps with bilateral support, less than 8 m, using a wheelchair for most activities) in 6 persons.

Sixteen participants (6♀, 10♂, 33.3 ± 9.3 years, AIS A-7, B-5, and C-4 individuals) performed EWT without SCES (Group 4, 12 training sessions), with similar inclusion/exclusion criteria and limitation for the level of spasticity MAS ≤ 3 (Modified Ashworth Scale). Most participants had a lesion at thoracic level (*n* = 12), three at thoracic-lumbar level, and one at low-cervical high-thoracic level with mostly unimpaired functions of the arms. The functional mobility of participants varied from 2 to 4 (3.1 ± 0.6, Rivermead Mobility Index), activities of daily living – from 40 to 90 (on average 63.4 ± 13.8, Barthel Index), and the Hauser Ambulation Index was estimated as 7 (7) and 8 (9) (Table 1).

### Walking in the Exoskeleton

Wearable exoskeleton ExoAtlet Global is designed to empower lower limb disable people to walk on level ground (European patent WO 2017/069652 A1, Berezij et al., 2017)^1^. The exoskeleton weighs 23 kg including battery and it bears its own weight by transferring the weight via its footplates to the ground. It is attached to the wearer at five main locations: footplate, shank, thigh, pelvis, and torso (Figure 1). Its shank, thigh and foot segment lengths and pelvic widths can be accommodated to different subject height/statures (weight up to 100 kg and height 1.55–1.95 m). Footplates are made of carbon fiber to host human feet (Figure 1B) so that one degree of freedom (ankle dorsi/plantar flexion) is passively sprung with certain stiffness (150 Nm/rad). The control of the exoskeleton is initiated/performed via a PC tablet or a “smart crutch” for experienced users. The control system of the ExoAtlet is unique and allows different functions and control modes: it collects data from body angles, allows to set the height and length of the step, sitting, performing sit-to-stand, standing still, stepping in place, level walking with different cycle durations, walking on angled surface, stepping over obstacles, and comfortable walking up and down stairs (Berezij et al., 2017; Pais-Vieira et al., 2020). Comparative characteristics of the exoskeleton ExoAtlet can be found in a review (Onose et al., 2016). In each lower limb, hip flexion/extension and knee flexion/extension are powered by actuators, providing appropriate kinematics of joint angle motion. The walking trajectories during the swing phase (reference joint angles) were defined based on ensemble-averaged walking patterns of 8 neurologically intact individuals during normal overground walking at the natural self-selected speed, recorded by means of a 16-cameras Vicon system at 120 Hz (Oxford, United Kingdom) (Berezij et al., 2017). The swing phase durations were similar across conditions, however, other parameters could vary in order to allow the SCI participants to incrementally increase their performance with training by selecting shorter cycle durations and/or longer stride lengths. Hip and knee flexion angles were decreased (scaled) during swing (relative to the reference trajectories recorded in neurologically intact individuals) to ensure more stable walking of paralyzed individuals, so that the selected program for stepping determined the kinematics of the Exo-gait with three different stride lengths (about 0.6, 0.75, and 0.9 m) and three different cycle durations (3, 4, and 5 s). Sensors built into each motor provide an automatic arrest of stepping if excessive torques (exceeding by 40% the reference hip or knee joint torques during walking in the exoskeleton in neurologically intact individuals) are detected (Berezij et al., 2017). The reference hip/knee joint torques were defined based on ensemble-averaged joint torques of neurologically intact individuals walking in the exoskeleton, recorded by means of torque sensors of the exoskeleton (Berezij et al., 2017). The exoskeleton was designed to walk with crutches (since it cannot provide full balance), which can bear a significant portion of body weight since SCI individuals use their upper limbs and body to assist foot loading and stepping. The design also included two rear handholds for one or two assistants for physical help and/or safety (typically used in the initial sessions but not for advanced pilots, see Supplementary Videos). An example of walking in the exoskeleton in the SCI subject before and after EWT with SCES is illustrated in Figure 1B and Supplementary Videos.

### Electrical Stimulation of the Spinal Cord

The stimulation method is thoroughly described in our previous studies (Shapkova and Schomburg, 2001; Shapkova, 2004). SCES was applied to the level of the mid-lumbar cord using the Viking Select stimulator (United States), the pair of conductive self-adhesive electrodes (3 cm× 4 cm) being placed on the skin over the Th12 vertebra (cathode) and centrally on the abdomen (anode) (Figure 1C). Once electrodes were mounted, the participant was placed in the supine position to determine the intensity of SCES. The magnitude of SCES (0.5 ms monophasic square-wave pulses) was about 1.3–1.4 of motor threshold in leg muscles. To determine the motor threshold, the electromyographic (EMG) activity was recorded bilaterally in 4 muscles (RF, rectus femoris; BF, biceps femoris; GL, gastrocnemius lateralis; TA, tibialis anterior), and the stimulation intensity was adjusted using 3 mA increments until a response was observed in at least 4 muscles (in some SCI individuals, not all muscles could be activated, see section “Results”). EMG activity was recorded at 2 kHz using bipolar surface electrodes (Nicolet Viasys Viking Select EMG System, United States). If leg muscle responses were low/absent or required too high amplitude currents (>70 mA), we considered the visible response of the abdominal muscles (multisegmental muscle responses were absent in two participants). In the anti-spastic mode, the magnitude of SCES was below the motor threshold. All SCES-procedures were painless. Some participants had previous experience of SCES and/or EWT (Table 1). We grouped participants according to the frequency of SCES (Table 1) and below we summarize the experimental procedure.

For our tasks, we set the frequency of SCES to 1 (Group 1), 3 (Group 2), and 67 (Group 3) pulses/s. In addition, in the control group (Group 4), we performed EWT without SCES. A general scheme of participant groups is illustrated in Figure 1A. First (in the preliminary study), in Group 1 of participants (Figure 1A and Table 1), with SCES at 1 pulse/s, we evaluated the compatibility of EWT and SCES in the context of potential disturbing influences and benefits, taking in account its strong influence on excitability of motor neurons and potential disturbing effects on body balance. Indeed, electrical stimulation *per se* may evokes perceptual or mechanical disturbing effects (for instance, it stimulates erector spinae and abdominal muscles), as well as even low frequency stimulation at 1 Hz may facilitate motor responses (Shapkova, 2004; Solopova et al., 2014). The rationale for using SCES at 3 pulses/s (Group 2, main study, Figure 1A) was to activate the spinal pattern generator networks (Shapkova and Schomburg, 2001; Shapkova, 2004). This frequency has been shown also to be effective in evoking stepping in the decerebrate cat (Iwahara et al., 1992). However, in the first session, two participants from the preliminary study and two participants from the main study were unable to use the exoskeleton because of severe spasticity (MAS 4) since the exoskeleton arrested after a few steps. These participants were excluded from Group 1 and 2, and formed Group 3 (Figure 1A). In this group, to perform EWT, we applied an anti-spastic SCES at 67 pulses/s beginning from the second or third session. After 2–3 sessions with 67 pulses/s, we used an alternating (changing every 10 min) stimulation at 67 pulses/s (to suppress spasticity, Pinter et al., 2000; Hofstoetter et al., 2014) and 3 pulses/s (to activate spinal locomotor networks, Shapkova, 2004). Using this stimulation method, they were able to complete the whole training program (9–11 training sessions with a comparable session duration as in Group 1 and 2, Table 1) and were included in the analysis as Group 3.

### Exoskeleton Walk Training With SCES

The study included short (∼2 weeks) intensive rehabilitative period of exoskeleton walk training with transcutaneous electrical stimulation of the spinal cord. All subjects received daily 40 min SCES in the stationary (supine) position prior to the EWT session. All participants were clinically stable at the time of examination and capable of walking in the exoskeleton with crutches and with the assistance of physiotherapist. The novices begun the course with learning to wear the exoskeleton, to stand up and sit down, to stand with crutches, to step in place and to walk straight. In the initial sessions, they required assistance by two or three physiotherapists. The number of sessions needed for novices to achieve independent stable walking with the exoskeleton was about 3–6 (Table 1). When a participant demonstrated stable walk with one assistant for safety (from the 3–6 session for novices, and from the first or second session for experienced exoskeleton walkers), electrical stimulation of the spinal cord was applied during EWT. On average, the total number of sessions over the 2 weeks of training was 7–15 depending on the subject, and the total duration of walking in the exoskeleton was ∼250–300 min. Participants in each group received about 8 sessions (range 6–10) of combined EWT and SCES (Table 1). The total session duration (wearing the exoskeleton), the duration of walking and the maximal non-stop walk duration per each subject are described in Table 1.

### Neurological Examination and Biomechanical Measurements

Spinal cord injury participants were admitted to the hospital for the purposes of this study and were submitted to neurological evaluation, routine radiological and neurophysiological tests. Radiological tests included available neuroimaging techniques of the spine and the spinal cord (CT, MRI). Neurological examination was performed before and after the training course using the American Spinal Injury Association Impairment Scale (AIS), spasticity – by the Modified Ashworth Scale (MAS), and ambulation was estimated using the Hauser Ambulation Index (HAI). Spinal motoneurons excitability was assessed by evaluating H-reflex responses (in the lateral gastrocnemius muscle of both legs), and the multisegmental muscle responses (in the rectus femoris, biceps femoris, lateral gastrocnemius, and tibialis anterior) to the electrical stimulus (0.5 ms) applied between vertebras Th11-12 (Emeliannikov et al., 2016). The GL H-reflex was evoked by stimulation of n. tibialis in popliteal fossa (0.5 ms). H-reflex and multisegmental muscle responses were recorded consecutively, without changing EMG electrodes position, on diagnostic complex Viking Select (Nicolet, United States). For the H-reflex, the peak-to-peak amplitude of the M-wave (over the 5–20 ms period after the stimulus) and the H-reflex (25–60 ms after the stimulus) was calculated from each sweep (Solopova et al., 2003; Emeliannikov et al., 2016). Using 5 mA increments, the stimulation intensity was incrementally adjusted from 5 mA to ∼100 mA and the H_*max*_/M_*max*_ ratio was also computed.

The vertical component of the ground reaction force was recorded in 13 participants by means of in-shoe sensors using the F-Scan System (Tekscan, United States) in two circumstances: the first time when the participants were able to perform stable exoskeleton-induced walking with one assistant for safety and second time after 6–7 sessions of training in the presence of SCES. The individual sensor elements of the F-Scan System were elastic and arranged in a matrix insole. The local vertical force sensed by each element was recorded at 100 Hz. The insole was interposed between the participant’s foot and the sole of the shoes. Before each trial, the mean level of each sensor was measured while the foot was unloaded (lifted) for 3–5 s and this value was used as a zero level. A measure for asymmetry index (ASI) (Kim and Eng, 2003; Aruin and Kanekar, 2013) was used to assess the GRF right-left symmetry (absolute value) between the participant’s left (L) and right (R) limbs:

ASI = abs(L-R)/[0.5 × (L + R)] × 100% (1)

An ASI = 0 represents perfect symmetry. The max vertical feet loading (normalized to the body weight and averaged across strides, left and right side being pooled together) was also calculated.

Effects of EWT (with and without SCES) were assessed based on the results of neurological examination at the beginning and at the end of course (performed by an independent neurologist, not involved in the study). Due to unique individual variability, idiosyncrasies and the relationship between clinical factors and the extent to which rehabilitation potential is realized, some additional method details for individual subjects are provided in the Results. The descriptive statistics included means and standard deviation of the mean (SD). Repeated measures analysis of variance (RM ANOVA) was used (after determination that the data were normally distributed, Kolmogorov-Smirnov test) to assess the effect of EWT with SCES on changes in the foot loading force when walking in the exoskeleton with crutches. Given the heterogeneity of the sample and the treatments given, we mostly showed averaged data and we indicated individual neurological scores for all participants and groups. The characteristics of participants, neurological evaluation and durations of training are described in Table 1.

## Results

### Learning to Walk in the Exoskeleton

While experienced exoskeleton walkers received SCES starting with the first or second session, novices required several sessions of training with the exoskeleton before we could apply SCES. After positioning the SCI participants in the upright posture, first, they were asked to learn to perform stepping in place movements and maintain balance while alternatingly loading the crutches with their upper limbs. After learning stepping in place movements, they were asked to walk forward along a 30-m walkway and could incrementally increase their performance with training. Specifically, we started EWT with the cycle duration 4–5 s and the shortest stride length (∼0.6 m). If the participant succeeded with stepping, we asked whether it would be comfortable for him/her to walk faster (i.e., to decrease the stride duration to 4 and 3 s and increase the stride length to ∼0.75 or 0.9 m) and, accordingly, we incrementally increased the walking speed throughout the course of 2-weeked EWT. Typical total duration of the experimental session was ∼1 h (Table 1). All subjects showed strong motivation since the first trial and throughout testing.

While the kinematics of Exo-gait was determined by the appropriate control of the exoskeleton actuators (Berezij et al., 2017), its functioning also depended on the interaction between the exoskeleton program and activity of a pilot, who could “follow” or “resist” stepping performance. The exoskeleton contained multiple force sensors and the control program provided an automatic arrest of walking if excessive torques were detected. In this case, the control of the exoskeleton changed to the posture maintenance mode (fixed joint angles) preventing the subject from falling. Thus, there was a period of “learning” for the subject to produce a minimal influence on the robot and SCI participants first performed standard training of walking in the exoskeleton (without SCES) to achieve stable gait. In fact, the duration of non-stop walk was shorter in the first sessions with respect to the final sessions (see below). All SCI participants were able to walk in the exoskeleton with crutches and/or assistance of two or three physiotherapists in the initial sessions. When they accomplished relatively stable walking with one assistant for safety (after a few training sessions), SCES was applied during EWT in all remaining sessions. The participants of Group 3 accomplished walking in the exoskeleton only using an anti-spastic SCES at 67 pulses/s (or an alternating, changing every 10 min, stimulation at 67 pulses/s to suppress spasticity and 3 pulses/s to activate spinal locomotor networks).

### Foot Loading in SCI Individuals During Walking in the Exoskeleton

**FIGURE 2:**
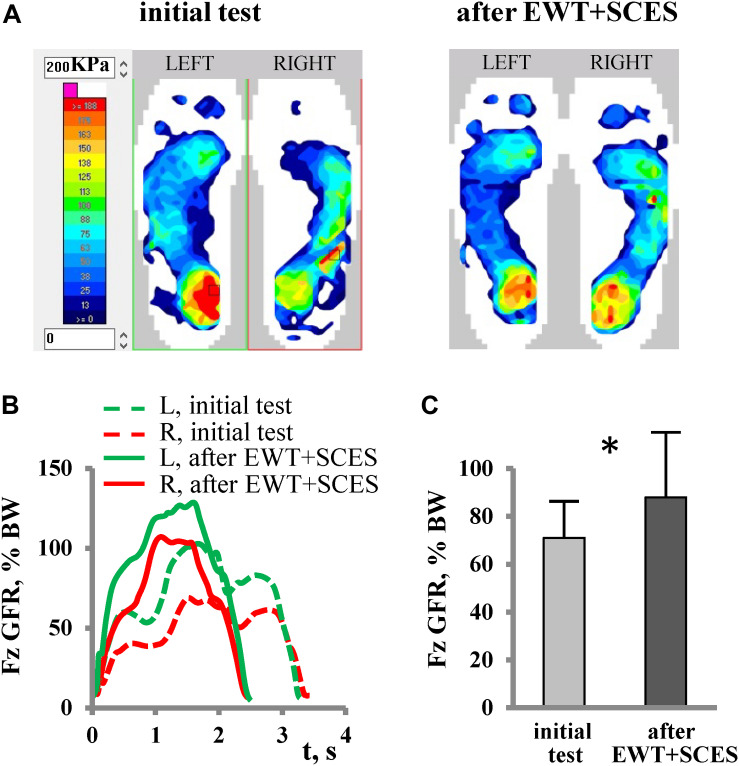
Foot loading characteristics. **(A)** An example of plantar pressure recordings at the end of the stance phase (during maximum foot loading of the right leg and of the left leg) in subject P5 (AIS A, fracture at Th10-12, 6 years post-trauma) during the initial test (2nd session, left panel) and after EWT with SCES (8th session, right panel). **(B)** Vertical component of GRF (Fz) expressed in% of body weight (BW) during the stance phase (averaged across 12 strides) in the same subject. **(C)** Max vertical foot loading force across all recorded subjects (mean + SD, *n* = 13, left and right foot data were pooled together) during the initial test and after a short course of EWT with SCES (asterisk denotes significant difference, RM ANOVA, *F* = 16.2, *p* < 0.001, *n* = 13). Note more symmetrical loading of both feet **(A)** and a significant increase in the foot loading **(C)** following EWT with SCES.

Spinal cord injury participants achieved the control of balance holding the crutches, as well as being assisted by the physiotherapists in the initial sessions (see Supplementary Videos). During Exo-walking, the body weight is distributed between the feet and crutch support. The beginners rely more on crutches, whereas with experience the participants increase loading on the feet. An example of foot pressure recordings is illustrated in Figures 2A,B for subject P5 (AIS A). Note a significant increase in foot loading following EWT with SCES (Figures 2A,B). Local loadings of the plantar side of the foot were variable for different subjects. The coefficient of variation in the max foot loading across strides was similar before [7.5 ± 2.6% (mean ± SD across subjects)] and after (6.9 ± 2.5%) the training course though the GRF right-left asymmetry index (ASI) decreased from 25 ± 16% (range 3–60%) to 15 ± 9% (range 3–33%). However, overall, there was a significant increment of the max feet loading after 2 weeks of exoskeleton walk training with SCES across all subjects (RM ANOVA, *F* = 16.2, *p* = 0.001, *n* = 13, Figure 2C), likely related to a lesser support of the body weight by crutches and increments in the walking speed/cadence after training.

The time course of the net vertical ground reaction force varied across participants, from a simple bell-shaped trajectory (with the timing of the maximum force around midstance, Figure 2B) to a two-peaked profile with the two maxima at the beginning and the end of stance. The latter profile has an apparent similarity with the shape of the vertical GRF force curve during normal over-ground walking or at moderate levels of body weight support in neurologically intact individuals (Ivanenko et al., 2002), but it cannot be interpreted in the same way because of the extra support by crutches and/or a more “passive” nature of foot loading forces in SCI individuals (e.g., a lack of the power in the ankle joint extensors at the end of stance). For instance, the distribution of foot pressure at the end of the stance phase in most paraplegic subjects showed typical loading on both forefoot and heel areas (Figure 2A), while the anterior-posterior center-of-pressure excursion is substantial in neurologically intact individuals during stance and results in merely forefoot loading at push-off (Ivanenko et al., 2002).

### Facilitation of Exoskeleton-Induced Walking and the Effect of Training

**FIGURE 3:**
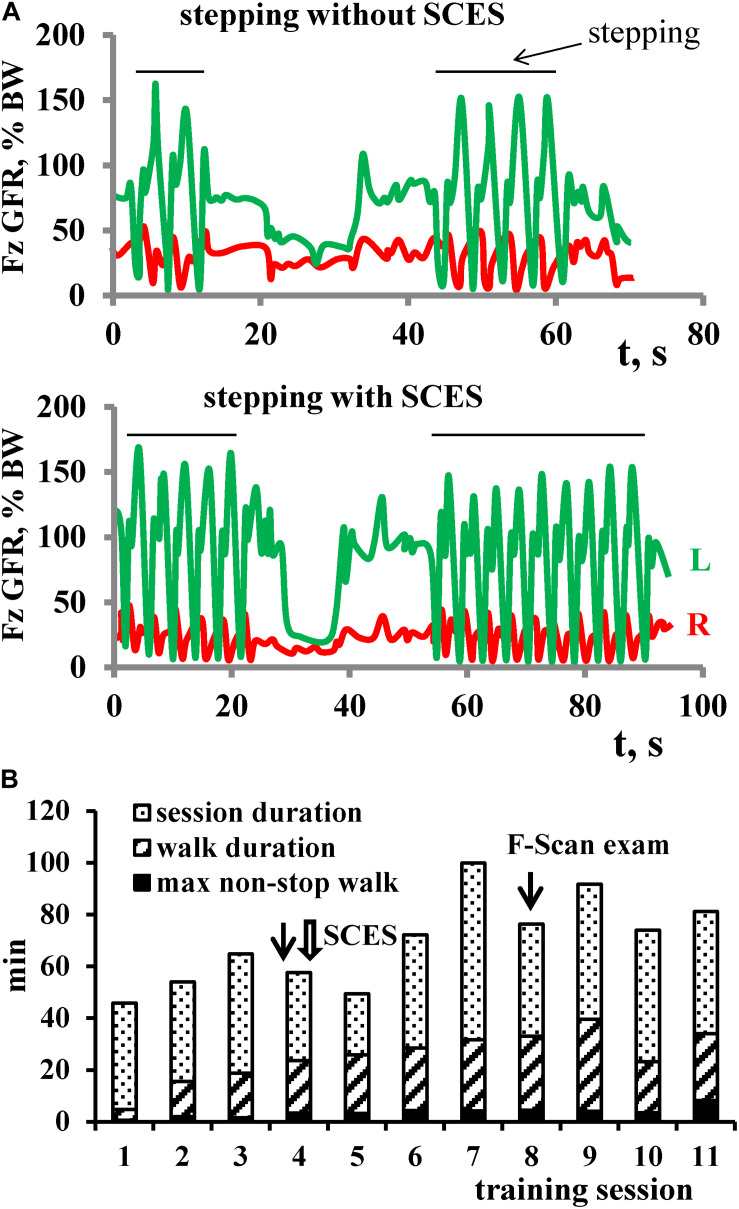
Facilitation of stepping with SCES (in the anti-spastic mode) in subject P16 with severe spasticity (AIS B, MAS 4). **(A)** Examples of vertical GRF in the 4th session of EWT (1st session with SCES) without (upper panel) and with (lower panel) SCES. Each panel shows 2 attempts of stepping in the exoskeleton (indicated by horizontal lines), corresponding to the initial and final series of alternating foot loading, respectively. Note more steps performed with SCES. **(B)** Diagram of EWT across 11 sessions: session duration (column as a whole), total duration of walking (oblique stripes) and maximal non-stop walk duration (black). Session (#4), in which SCES started, is marked by a wide arrow. Sessions, in which F-Scan recordings were performed, are marked by thin arrows.

The most evident effect of SCES on stepping was observed in Group 3 (MAS∼4). In participants with severe spasticity, it was typically difficult to start the training session since the exoskeleton automatically stopped after a few steps due to an excessive resistance (Figure 3, upper panel). In two participants, muscle spasms occurred during the sit-to-stand transition before the Exo-walk started. To prevent this, the participants first donned the exoskeleton in the supine position and then we placed them directly to the upright position avoiding a sit-to-stand maneuver. The application of SCES in the anti-spastic mode (67 pulses/s) immediately increased the number of Exo-steps (Figure 3A, lower panel). With training, the duration of the non-stop step sequences gradually increased (Figure 3B). In the first session with SCES, subject P16 increased the maximum non-stop duration from 22 to 50 strides and performed 122 strides at the end of the training course. Subject P19 increased the max non-stop duration from 34 to 121 strides beginning from the 3rd session and performed 284 strides (14 min) already in the 4th session. Subjects P17 and P18 performed up to 32 and 40 min of non-stop walking at the end of EWT.

Overall, the average duration of walking in Group 3 did not differ significantly from Group 1 and 2 (Table 1). The max non-stop duration in the Groups 3 and 2 was comparable (23.6 ± 15 min vs. 28.3 ± 8.2 min) and exceeded that of the Group 1 value (10.6 ± 6.7 min). Thus, application of SCES in the anti-spastic mode allowed individuals with severe spasticity to walk in the exoskeleton, and the amount of training within the course was comparable to other groups. Furthermore, it is also worth noting that, in all participants of the Group 3, the level of spasticity decreased after EWT with SCES (Table 1).

**FIGURE 4:**
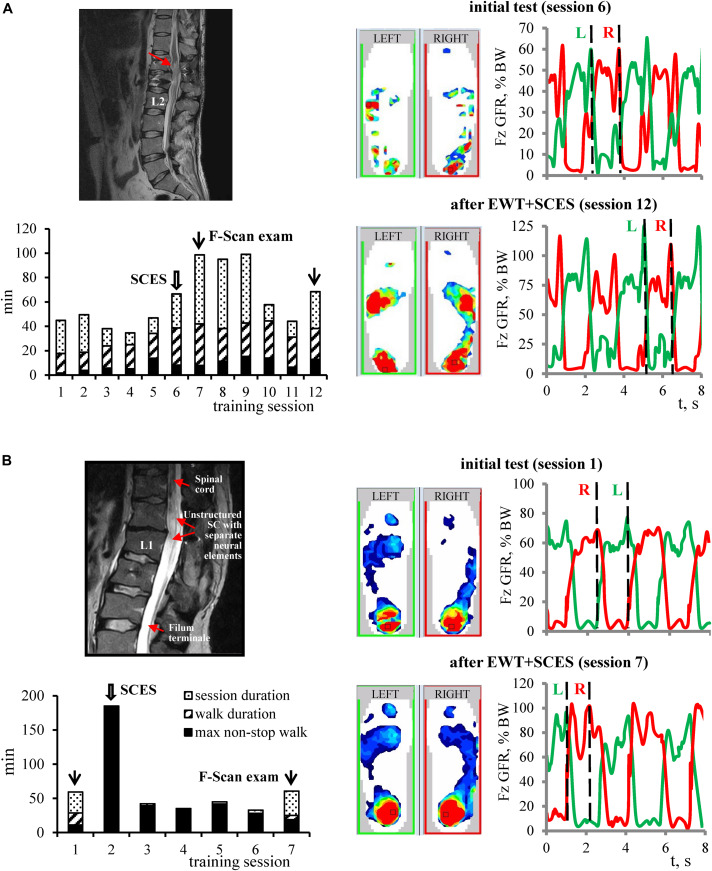
Examples of changes in the foot pressure characteristics during EWT with SCES in subjects with motor incomplete **(A)** and complete **(B)** paraplegia. For each example: MRI image, diagram of EWT (same format as in Figure 3B), plantar pressure recordings and vertical foot loading forces (right) during the initial test and after EWT with SCES are shown. Vertical dashed lines on the right panels correspond to the time when plantar pressure distribution patterns are illustrated on the middle panels (corresponding to the maximum foot loading at the end of the stance phase). **(A)** Subject P4 (AIS C, 2.5 years post-trauma). MRI: structural changes of the spinal cord with post-traumatic gliosis. After 6 sessions of training, there was a twofold increase of the plantar pressure and the amplitude of GRF. Also, P4 subject increased an ability to walk over-ground using a walker for balance (without exoskeleton) from 3 to 40 strides. The subject noted the emergence of paresthesia in leg muscles and new “feeling of support” with an increase of sensation (by + 3 AIS score for both light touch and pin-prick). **(B)** Subject P12 (AIS B, motor – 3, sensory 103/103, 11 years post-trauma), advanced EXO-walker. MRI: non-structured spinal cord at the level for lumbar enlargement (Th12-L1) with separated neural elements. Complicated L1 vertebral fracture with SC compression. Surgery: SC decompression and instrumental fixation. P12 is actively engaged in sports, as an actual member of the National Russian Paralympic Team in curling. In the first training session with SCES, the subject improved his achievement of non-stop walking in the exoskeleton from 120 min (previous achievement) to 185 min and set the new record. After 6 sessions of training, the pressure under the feet increased and spread to the front part of the foot, and the amplitude of GRF increased from ∼75% BW up to ∼100–105% BW. Wide arrows – sessions in which SCES started, and thin arrows – sessions in which F-Scan recordings were performed.

The positive effect of training was observed in both motor incomplete and complete paraplegic subjects. Figure 4A illustrates a diagram of training (lower left panel) and an example of changes in the foot pressure characteristics during EWT with SCES (right panels) in subject P4 with incomplete paraplegia. After 6 sessions of training, there was a twofold increase in the amplitude of GRF. Also, after EWT with SCES, P4 subject increased an ability to walk over-ground using a walker for support and balance (without exoskeleton) from 3 to 40 strides. The subject noted the emergence of paresthesia in leg muscles and new “feeling of support” with an increase of sensation (by + 3 AIS score for both light touch and pin-prick, Table 1).

All participants of the Group 1 and 2 also reported facilitation of exoskeleton walking when SCES was applied. The participants of the Group 1 (SCES at 1 pulse/s) attributed this facilitation to improvements in the sensitivity of the body below the lesion. Group 2 participants (SCES at 3 pulses/s) noted an ability to walk for a greater distance and/or with less fatigue. The latter could be illustrated in subject P12 (Figure 4B). P12 is actively engaged in sports, as an actual member of the National Russian Paralympic Team in curling. In the second training session with SCES, this participant improved his achievement of non-stop walking in the exoskeleton from 120 min (previous achievement) to 185 min and set the new record. After 6 sessions of training, the pressure under the feet increased and spread to the front part of the foot, and the amplitude of GRF increased from ∼75% BW up to ∼100–105% BW (Figure 4B). Also, at the end of EWT, his AIS motor scale increased by 3 points (from 6 to 9, Table 1).

### Excitability of Spinal Motor Neurons

**FIGURE 5:**
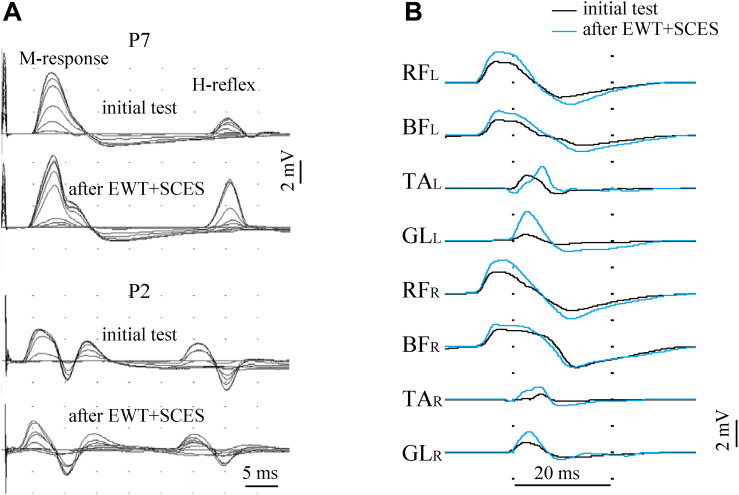
Excitability of lumbar motor neurons before and after the course of EWT with SCES assessed by the presence/amplitude of the H-reflex [**(A)**, superposition of 12 records with gradually increased stimulus magnitude in two subjects] and by the evoked multisegmental muscle responses to supramaximal electrical stimulus at the level of Th11-12 vertebrae **(B)** in subject P7 (AIS A, 2.5 years post-injury). The H-reflex was evaluated in the lateral gastrocnemius muscle and was illustrated for two participants **(A)**: with an initially low H_*max*_/M_*max*_ ratio (subject P7, upper panels) and with an initially high H_*max*_/M_*max*_ ratio (subject P2). After the 2 weeks of training, the H-reflex significantly increased in P7 and decreased in P2 (a kind of “normalization” toward an optimal 0.4–0.6 value for the H_*max*_/M_*max*_ ratio). RF, rectus femoris; BF, biceps femoris; TA, tibialis anterior; GL, gastrocnemius lateralis (R, right; L, left).

Excitability of the spinal motor neurons was assessed by the presence/amplitude of H-reflex responses and by the evoked multisegmental muscle responses to supramaximal electrical stimulus at the level of Th11-12 vertebrae. Figure 5 illustrates an example of such responses before and after EWT with SCES. Excitability of the spinal motor neurons below lesion was observed in most SCI participants. In some subjects we failed to observe muscle responses: M- and H-responses in the lateral gastrocnemius muscle were not detected bilaterally in 2 subjects and the H-response was not found in 5 subjects (bilaterally in 4 subjects and unilaterally in 1 subject). After the training period, we detected the M-response in one of those subjects and the H-response in another one. The magnitude of the H-reflex varied within 0.1–3.2 mV across all subjects, and the H_*max*_/M_*max*_ ratio – within 0.07-1.0. In 6 participants with an initially low H_*max*_/M_*max*_ ratio, we observed an increase of this ratio by more than 30% (Figure 5A) and a decrease in 3 subjects with an initially high ratio. Thus, there was a kind of “normalization” of this parameter toward an optimal 0.4–0.6 value after the 2 weeks of training.

Multisegmental muscle responses to the electrical stimulus applied between vertebras Th11-12 allowed to assess the excitability of multiple motor pools (we recorded EMG responses in 8 leg muscles) and they varied across muscles and subjects (0.1–8.5 mV). Multisegmental muscle responses were recorded in 17 subjects and were absent in two participants. They could be evoked in all (14/19) muscles or in the part (3/19) of them, and the number of muscles with evoked responses was the same before and after training. Not all changes in the excitability of different motor pools of the lumbosacral enlargement could be clearly interpreted because of technical reasons (due to some differences in the skin impedance and/or electrode placement). Nevertheless, both methods of the neurophysiological examination showed the excitability of at least a part of motor neurons of motor pools in most SCI subjects.

### Changes in the Neurological State

During the course of EWT with SCES, 15 out of 19 participants reported occurrence and subsequent strengthening of paresthesia in leg muscles, new non-differential feeling of passive motion in leg joints and a “sense of support.” In one subject (P4), paresthesia reached the level of pain, interfering with night’s sleep and requiring medication using non-steroidal anti-inflammatory drug injections. Nevertheless, at the request of the subject, EWT with SCES was continued and 2 days later the subject reported an overhauled feeling of “whole legs” instead of mosaic sensations in some parts of the thigh and foot.

**FIGURE 6:**
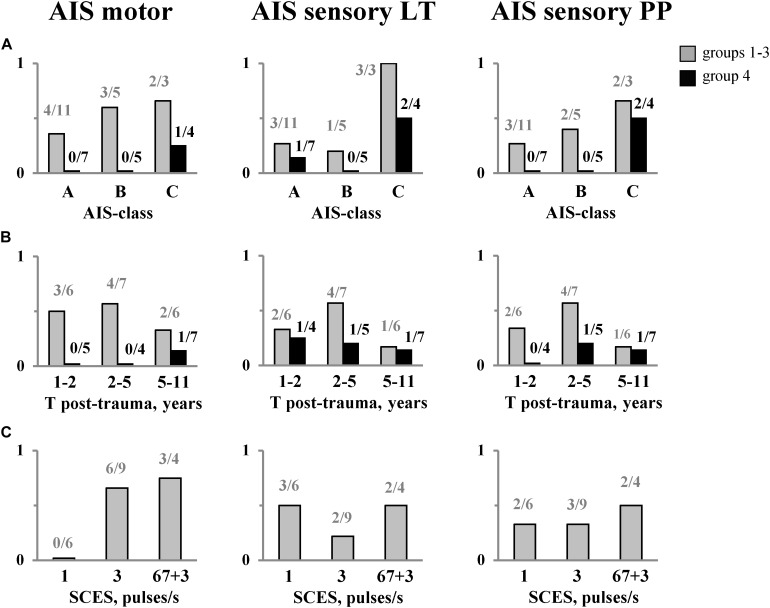
The proportion of subjects with neurological improvements in motor and sensory (LT &PP) AIS after EWT with SCES depending on: completeness of spinal cord damage **(A)**, time after injury **(B)**, and frequency of SCES **(C)**. The number of subjects is also indicated. The data for the group 4 (EWT without SCES) are shown separately and marked in black. LT, light touch; PP, pin-prick. Note a significantly smaller proportion of subjects in group 4 showing improvements in the neurological indicators [especially in SCI **(A,B)**] with respect to the groups with SCES [**(A)**, see also Table 1].

Table 1 shows overall changes in the neurological state (AIS, MAS) and locomotor ability (HAI) in all SCI participants after EWT with SCES, and Figure 6 illustrates the proportion of participants with neurological improvements in motor and sensory AIS depending on completeness of spinal cord damage (ASIA-class), time after injury, and frequency of SCES. Neurologic examination at the end of the training course showed an increase in pain sensitivity in 8 subjects, tactile sensitivity in 7 subjects and the leg muscle strength in 9 subjects (Table 1). After training we obtained some motor or sensory improvements in all AIS-C subjects (3/3) and in more than a half of AIS-A (7/11) and B (3/5) subjects (Figure 6A). There was no clear dependence of the rate of neurological progress on the time after injury (Figure 6B), though the progress in motor and sensory AIS seemed to be more often observed in the participants during the period of 2–5 years after injury.

In Group 1 (stimulation at 1 pulse/s), there were no improvements in the motor scale (Figure 6C, left panel), while in Group 2 (3 pulses/s) and Group 3 (“67 + 3” pulses/s), the proportion of individuals with improvements in the AIS motor scale was comparable (6/9 and 3/4, respectively). After the training course, 8 out of 19 subjects improved the Hauser Ambulation Index (Table 1). Two participants (AIS C), who were initially unable to walk, started to walk using walkers and stopped using a wheelchair when ambulating indoors.

In Group 4 (EWT without SCES), we also observed increments in foot loading with training (68 ± 10% BW before and 89 ± 11% BW after training). However, a substantially smaller proportion of individuals showed improvements in the AIS motor and sensory scales (especially in SCI-A and SCI-B participants) after the course of training with respect to the groups with SCES (Figure 6A, see also Table 1).

## Discussion

In this study, we evaluated the compatibility of EWT and tonic electrical stimulation of the lumbar enlargement and explored the main effects of 2-weeked combined SCES and EWT. We explored diverse stimulation parameters and tested 35 individuals with post-traumatic impairment of the spinal cord (Table 1). The results demonstrated that SCES and EWT are well compatible, and SCES may facilitate training and walking in the exoskeleton in SCI individuals by activating the locomotor networks and augmenting compensative sensitivity. Facilitation of stepping with SCES was noticed by all participants, but the nature of such effects requires further investigations.

### Benefits of Combined EWT and SCES

There might be several benefits of EWT with a non-invasive SCES in regard to locomotor training on a treadmill with a body weight support. Stepping in the exoskeleton provides a unique opportunity to experience over-ground weight bearing stepping. Moreover, it allows to use the upper body and arm muscles to assist leg movements and to challenge balance control by coordinating the movements between the arms, trunk and lower limbs, thus promoting connections between lumbosacral and cervical enlargements (Sylos-Labini et al., 2014; Gad et al., 2017). Reflex-related activity of lower limb muscles innervated from the spinal segments below the lesion during assisted walking in the exoskeleton (Sylos-Labini et al., 2014; Gad et al., 2017) might also be beneficial for potential gait rehabilitation since there is a relationship between facilitation of segmental reflexes and the ability to recover gait (Dietz et al., 2009; Thompson and Wolpaw, 2014). SCES aims at activating the spinal locomotor networks below the lesion, changing them to a physiologically active state and reinforcing synaptic connections of the spinal pattern generation circuitry and supraspinal-spinal connectivity (Shapkova, 2004; Gill et al., 2018; Hofstoetter et al., 2018; Wagner et al., 2018). Thus, in addition to gait assistive aspects of exoskeleton robotic devices in severely paralyzed individuals, the proposed approach of EWT with SCES may also be beneficial for gait rehabilitation.

After the training course of 2 weeks, the SCI individuals, initially able to ambulate with walkers, increased the walking distance but did not stop to use a wheelchair. The ability to walk 20–30 using a walker for balance did not change significantly their life. However, two participants, who were initially unable to walk, started to walk using walkers and stopped to use a wheelchair indoors. In our understanding, it was a drastic change in their lifestyle and future quality of life.

After 2 weeks of intensive training, we obtained some motor or sensory improvements in all AIS-C subjects (3/3) and in more than a half of AIS-A and B subjects (10/16). We did not find a dependence of the rate of neurological progress on the time after injury (within 1–11 years, Figure 6B). A traditional view and prognosis of chronic SCI is based on extensive atrophy in the spinal circuitries distal to the lesion (Dietz and Müller, 2004) and on limited plasticity and functional recovery after more than a year after a spinal complete lesion. Our findings may challenge a traditional prognosis for chronic SCI. Finally, improvements in the neurological scores were substantially limited in the Group 4 (especially in SCI A and B patients) with respect to the groups with SCES (Table 1 and Figure 6). Nevertheless, since multiple factors might play a role, further investigations are needed to develop personalized neuromodulatory interventions to meet the specific needs of individuals with spinal cord injury (James et al., 2018).

### Frequency-Dependent Effects of SCES

The interventions tested included EWT with non-invasive spinal cord stimulation at 1 pulse/s (Group 1, Table 1), 3 pulses/s (Group 2) and high-frequency anti-spastic stimulation (Group 3). The most evident is an anti-spastic effect of SCES at 67 pulses/s. The usage of the spinal cord electrical stimulation to relieve spasticity dates back to the end of seventies (Richardson and McLone, 1978; Siegfried et al., 1978; Vodovnik et al., 1984) and is associated mostly with epidural stimulation (Hunter and Ashby, 1994; Midha and Schmitt, 1998; Pinter et al., 2000). It has been shown that epidural stimulation with the electrodes placed over the lumbar posterior roots in the frequency range of 50 Hz–100 Hz can significantly suppress severe lower limb spasticity (Pinter et al., 2000). The effect was accounted for by changes in the excitability of neural circuits in the lumbar spinal cord through continuous posterior root activation. Technologies with the implantation of anti-spastic stimulants have demonstrated moderate efficacy as compared to their high cost and did not find a widespread application. Nowadays, after obtaining evidence of a comparable effect of epidural and percutaneous electrical stimulation (Hofstoetter et al., 2018), an interest in the anti-spastic electrical stimulation has been revived (Hofstoetter et al., 2014). Despite some technical differences between our method and the one described by Hofstoetter et al. (2014) in the location of the electrodes and stimulation frequency (67 vs. 50 pulses/s), both methods are based on the same principle and produce similar anti-spastic effect. The application of SCES in the anti-spastic mode had an immediate effect on the number of non-stop steps and facilitated stepping in the exoskeleton (Figure 3), with some improvements in the motor and sensory AIS after 2 weeks of training (Figure 6C).

Spinal cord electrical stimulation at 1 pulse/s is routinely used in our clinic to increase excitability of motor neurons in flaccid paralysis. In this study, the frequency of 1 pulse/s (Group 1, Table 1) was chosen to test a possible disturbing effect of SCES on the exoskeleton-induced walking. Despite the magnitude of stimulation exceeded the motor threshold and the sensitivity to stimulus increased with the number of sessions, no subject reported any disturbances associated with SCES. The subjects noticed “the returning feeling of the lower body or the whole leg instead of the part of it” and a new feeling of ground support. Subjective impressions of participants were supported by the results of the neurological examination: in Group 1, improvements in sensitivity were found more often as compared to Group 2 (Figure 6C, middle panel). Based on these observations, we suggest that such stimulation gives a greater impact on sensitivity rather than results in improvements in the motor scale (Figure 6C).

For EWT with SCES at 3 pulses/s, we assumed to activate the spinal locomotor networks and/or potentiate their activity (Shapkova and Schomburg, 2001; Shapkova, 2004). It is also worth noting that this frequency was found to be most effective in inducing locomotion in the decerebrate cat (Iwahara et al., 1992). The experienced exoskeleton-walkers reported that with 3 pulses/s SCES they were able to walk longer without fatigue. For instance, potentiation of the locomotor ability was clearly demonstrated by the subject P12. This advanced exoskeleton-walker significantly improved his best achievement for the non-stop walk duration in the exoskeleton in the 2nd training session of EWT with SCES (Figure 4B). At the end of the training course, we also found significant changes in the F-Scan recordings (Figure 4B) and an increase of the muscle strength (by 3 points in the AIS motor score, Table 1). Taking into account the high physical conditions of P12 (active member of the National Paralympic Team) and 3-year experience with EWT, we explain such effects by the efficacy of SCES in activating spinal neural structures and/or increasing their excitability. Overall, in the subjects receiving 3 pulses/s SCES, an increase in the muscle strength was observed more often than in the Group 1 (Figure 6C).

### Limitations of the Study

The results of the study are encouraging but need to be confirmed in a larger SCI population, also because the effects may be heterogeneous due to multiple clinical or methodological factors. For instance, we used SCES during both stationary (supine) condition and during EWT so that its duration was overall twice longer as compared to the EWT duration. We performed SCES in the stationary condition to activate the locomotor networks and thus to reinforce the potential effect of combined training but we do not know its relative effect. Also, there might be subject-specific or condition-specific optimal parameters of SCES. Various techniques have been developed to stimulate the central pattern generators (CPGs) for stepping, which may include both diffuse and quite specific tuning effects, although tonic and rhythmic spinal activity control are not separate phenomena but are closely integrated to properly sustain CPG functioning (Hofstoetter et al., 2017; Ivanenko et al., 2017). For instance, differential spinal cord segment stimulation (epidural or transcutaneous) is a promising tool to restore the functioning of the CPG circuitry in both animals (Capogrosso et al., 2016; Wenger et al., 2016) and humans (Solopova et al., 2017; Angeli et al., 2018; Gill et al., 2018; Wagner et al., 2018), and such stimulation techniques might take into account differential activation of spinal segments to selectively drive neuroprostheses depending on walking conditions (Dewolf et al., 2019) and individual affordability and efficacy of such approaches (James et al., 2018).

Nevertheless, the activation of CPG circuits largely depends on the presence of a sustained tonic excitatory drive, as it can be elicited by electrical spinal cord stimulation, or by specific pharmacological neuromodulation (Hofstoetter et al., 2017). In our study using tonic SCES, the subjective reports, increments in the max non-stop walk duration (Figure 3), the differential effect of the SCES frequency (Figure 6C left panel) and limited improvements in the neurological scores in the control group of subjects for EWT without stimulation (Figure 6), point to a facilitatory effect of stimulation. The findings also corroborate a general notion about a facilitatory outcome of SCES on the functioning of the locomotor pattern generation circuitry (Shapkova, 2004; Gill et al., 2018; Hofstoetter et al., 2018; Wagner et al., 2018), as well as the results of a recent case study that spinal cord stimulation enhances the level of effort that the subject can generate while stepping in the exoskeleton along with changes in autonomic functions including cardiovascular and thermoregulation (Gad et al., 2017). Also, no clinical changes were observed in the previous study on exoskeleton walk training in motor complete paraplegic individuals in the absence of concurrent SCES (Sylos-Labini et al., 2014), consistent with a limited effectiveness of robot-assisted gait training in severely paralyzed persons (Swinnen et al., 2010; Roy et al., 2012; Sale et al., 2012). Thus, EWT with SCES represents a considerable potential for gait rehabilitation after chronic spinal cord injury. The neurological improvement obtained after the short intensive course of combined SCES and EWT in chronic SCI individuals also suggests that a similar or greater improvement may be expected in acute paraplegic persons.

While improvements of stepping in the exoskeleton (Figures 2–4) and neurological improvements in the motor and sensory scales were observed (Figure 6), the course of 2 weeks is apparently too short and we have to find an optimal course duration and/or the number of courses to provide clinically significant and stable results for both acute and chronically paralyzed persons. Also, given that neurological improvements were observed in participants who previously received EWT or SCES interventions (Table 1), one could probably expect a cumulative effect of a series of courses.

## Conclusion

This study showed that percutaneous electrical stimulation of the lumbar enlargement and exoskeleton-induced walking are well compatible and provide walking assistance for SCI individuals. The exoskeleton assists both posture and leg movements, and SCES may facilitate training and walking in the exoskeleton in individuals with chronic SCI. The results also suggest that facilitation may be frequency dependent (3 pulses/s to activate the locomotor networks, 1 pulse/s to augment compensative sensitivity) though further investigations with a larger sample of participants are necessary to understand the benefits of training with different modes of stimulation and the underlying mechanisms. SCES in the anti-spastic mode can decrease spasticity and thus makes the exo-walk training possible for individuals with severe spasticity. The 2-week intensive synergistic effect of EWT with SCES enriched the locomotor ability and evoked neurological improvements in chronic SCI individuals, including complete paralysis, being beneficial for gait rehabilitation after SCI.

## Data Availability Statement

The datasets generated for this study are available on request to the corresponding author.

## Ethics Statement

The studies involving human participants were reviewed and approved by the Local Ethical Committee of the Saint-Petersburg State Research Institute of Phthisiopulmonology. The patients/participants provided their written informed consent to participate in this study.

## Author Contributions

ES organized and conducted the study, analyzed the clinical data, and wrote the draft of the manuscript. EP conceived and initiated the study and performed the biomechanical measurements and data analysis. DE conducted the study, performed the neurophysiological examination, and prepared illustrations. YI discussed the clinical and biomechanical data at all stages of the study, initiated the manuscript writing, and edited the manuscript.

## Conflict of Interest

The powered exoskeleton for this study was granted by its manufacturer “ExoAtlet”. EP is the major constructor of the “ExoAtlet” exoskeleton. The remaining authors declare that the research was conducted in the absence of any commercial or financial relationships that could be construed as a potential conflict of interest.

## Footnotes

1 https://www.exoatlet.com/en

## Supplementary Material

VIDEO S1 Video fragments of stepping in the exoskeleton of subject P4 – initial test.

VIDEO S2 Video fragments of stepping in the exoskeleton of subject P4 – after EWT with SCES. Written informed consent was obtained from the individual for the publication of these videos and recognizable image.
